# Medical Items

**Published:** 1892-02

**Authors:** 


### Medical Items.

Recent legislative enactments in Arkansas mention eight instances of unprofessional conduct which warrant the revocation of the physician's license. These are:

1. Procuring or aiding and abetting in the procuring of criminal abortion.

2. Employing or using what are known as cappers, steerers, or drummers, or the subsidizing of hotels or boarding-houses to procure practice.

3. The obtaining of a fee on the assurance that a manifestly incurable disease can be permanently cured.

4. The wilful betrayal of a professional secret to the detriment of a patron.

5. All advertisements of medical business in which untruthful and improbable statements are made.

6. All advertisements of any medicine or means whereby the monthly periods of women can be regulated or the menses re-established.

7. Conviction of any offence involving moral turpitude.

8. Habitual drunkenness.—*Med. Record.*

---

The College of Physicians of Philadelphia announces that the next award of the Alvarenga Prize, being the income for one year of the bequest of the late Senor Alvarenga, and amounting to about one hundred and eighty dollars, will be made on July 14, 1892. Essays intended for competition may be upon any subject in medicine, and must be received by the secretary of the college on or before May 1, 1892. It is a condition of competition that the successful essay or a copy of it shall remain in possession of the college. Charles W. Dulles, Secretary.

Dr. J. B. S. Holmes has associated with him at his Sanitarium, Dr. W. E. B. Davis, recently of Birmingham, Ala. Dr. Davis is president of the Tri-State Medical Society of Alabama, Georgia and Tennessee; secretary of the Southern Surgical and Gynecological Society; vice-president of the American Medical Association; member of the Birmingham Board of Medical Examiners; surgeon to the Birmingham Hospital of United Charities, and is one of the brightest, most prominent and most promising surgeons in the South. He has already made considerable fame for himself in gynecological and abdominal surgery.

---

Dr Hudson, class of '86, Atlanta Medical College, and second honor man, passed through Atlanta recently on his way to Europe. He will spend several months there and has promised to write monthly letters to THE JOURNAL upon interesting medical subjects. Knowing Dr. Hudson's ability we can promise our readers something excellent in these letters.

---

AT the next commencement of the Southern Medical College, March 3, an Alumni Association of the college will be organized, with permanent headquarters in Atlanta. It is hoped by those undertaking this work that all alumni of the college will be present, who can do so, to assist in the organization. Those who expect to come will please communicate with Dr. J. W. Price, West End, Atlanta, Ga.

---

ON January 14th, Dr. J. A. Childs, of Atlanta, was married to Miss Susie Pittman, also of Atlanta. THE JOURNAL extends to the happy pair its good wishes and congratulations.

---

THE *Therapeutic Gazette* is now to be edited by Drs. Hobart A. Hare, Geo. A. de Scheveinitz and Edward Martin, all of Philadelphia.

